# Combined use of companion planting and PGPR for the assisted phytoextraction of trace metals (Zn, Pb, Cd)

**DOI:** 10.1007/s11356-020-07885-3

**Published:** 2020-02-08

**Authors:** Agnieszka Konkolewska, Aneta Piechalak, Liliana Ciszewska, Nina Antos-Krzemińska, Tomasz Skrzypczak, Anetta Hanć, Krzysztof Sitko, Eugeniusz Małkowski, Danuta Barałkiewicz, Arleta Małecka

**Affiliations:** 1 Department of Biochemistry, Adam Mickiewicz University in Poznan, Uniwersytet Poznanski 6 Street, 61-614 Poznan, Poland; 2 Laboratory of Genome Biology, Adam Mickiewicz University in Poznan, Uniwersytet Poznanski 6 Street, 61-614 Poznan, Poland; 3 Laboratory of RNA Biochemistry, Adam Mickiewicz University in Poznan, Uniwersytet Poznanski 6 Street, 61-614 Poznan, Poland; 4 Department of Bioenergetics, Adam Mickiewicz University in Poznan, Uniwersytet Poznanski 6 Street, 61-614 Poznan, Poland; 5 Institute of Biology and Human Evolutionary, Adam Mickiewicz University in Poznan, Uniwersytet Poznanski 6 Street, 61-614 Poznan, Poland; 6 Department of Trace Analysis, Faculty Chemistry, Adam Mickiewicz University in Poznań, Uniwersytet Poznanski 8 Street, 61-614 Poznan, Poland; 7 Faculty of Natural Sciences, University of Silesia in Katowice, 40-032 Katowice, Poland; 8 Laboratory of Biotechnology, Institute of Molecular Biology and Biotechnology, Adam Mickiewicz University in Poznan, Uniwersytet Poznanski 6 Street, 61-614 Poznan, Poland

**Keywords:** Phytoextraction, Elements, Companion planting, Plant growth–promoting bacteria PGPR

## Abstract

Biomass production and metal accumulation in plant tissue (bioconcentration) are two critical factors limiting the phytoextraction rate. Metal translocation to aboveground organs should be accounted for as the third most important factor, as harvesting of the plant roots is usually economically disadvantageous. These three parameters could be potentially increased with the use of companion planting, a well-known agricultural technique, and inoculation with plant growth–promoting bacteria (PGPB). The aim of the study was to determine whether intercropping and inoculation with endophytic PGPB (*Burkholderia phytofirmans* PsJN^T^) can increase the efficiency of phytoextraction of Zn, Pb, and Cd. The study was conducted on *Brassica juncea* (L.) Czern. “Małopolska” grown in a monoculture or co-planted with *Zea mays* L. “Codimon” and *Medicago sativa* L. “Sanditi.” Results show that companion planting and inoculation with rhizobacteria can increase the efficiency of metal phytoextraction, mainly by increasing the yield of dry biomass and the survival rate of plants grown on contaminated soil. We have shown that the simultaneous planting of *B*. *juncea* with *M*. *sativa* and inoculation with PGPB were the most efficient variants of assisted phytoextraction reaching a recovery of 95% Zn, 90% Cd, and on average about 160% Pb compared with control *B*. *juncea* plants grown in monoculture.

**Electronic supplementary material:**

The online version of this article (10.1007/s11356-020-07885-3) contains supplementary material, which is available to authorized users.

## Introduction

Trace metals (such as Cd, Pb, Cu, and Zn) present in excess negatively affect plant growth, development, and biomass yield (Weyens et al. 2009; Andersen et al. 2018). After emission to the environment, these elements can enter the food chain through plants, to be later accumulated in higher levels of consumers, posing a threat to animal and human health (Aelion and Davis 2007; Bhattacharyya and Jha 2012; Douay et al. 2013). Contaminated soil can be remediated with phytoextraction, which uses the natural or induced capacity of plants to uptake and accumulate metals from the soil (Jadia and Fulekar 2009). It is considered a low-cost alternative compared with available methods of remediation (Sarma 2011).

Two main factors which limit the phytoextraction rate are biomass production and metal accumulation in plant tissue. Additionally, because harvesting of plant roots in the process is not economically feasible, another aspect—metal translocation to aboveground organs—should be considered as the third most important factor. Thus, in order to increase the efficiency of the process and make it economically viable, both biomass production and/or metal accumulation should be improved together with translocation to aerial parts.

Plant ability to take up and accumulate trace metals efficiently in the aboveground tissue is often expressed as a bioconcentration coefficient/factor (BCF), i.e., the ratio of metal content in the shoot tissue to the content in soil (McGrath and Zhao 2003). Robinson et al. (2015) estimated that a BCF = 14.8 of plants that produced 5 tons h^−1^ would be needed in order to decrease the contamination by 50% in a 25-year period; but if the plant produced 10 tons h^−1^, a BCF = 7.4 t only, for a soil contaminated with one metal to a depth of 20 cm at a soil density of 1.3 g × cm^−3^.

However, the selection of plants with an appropriate coefficient is not straightforward. Some plants endemic to soil enriched in minerals can accumulate high levels of metals. These so-called hyperaccumulators are characterized by a BCF coefficient of more than 1 (even reaching 50–100), whereas most plant species have a BCF factor for metals of < 1 (Ali et al. 2013). The main physiological mechanisms underlying the trait of hyperaccumulation are enhanced uptake in roots, efficient xylem loading, and increased detoxification levels (Verbruggen et al. 2009, 2013).

Higher metal accumulation can be also obtained in plants by stimulation, e.g., with chelators or microorganisms, the strains of which secrete substances that promote metal mobilization in soil (Vamerali et al. 2010; Wood et al. 2016; Sobariu et al. 2017). Endophytic bacteria have developed several types of mechanisms by which they reduce the toxicity of metal ions. These include the transformation of metal ions into less toxic forms and metal sequestration in extracellular and intracellular polymers as well as precipitation, adsorption, or biomethylation (Rajkumar et al. 2013). In addition, microbial inoculation may have other positive effects on plants: reduction of stress propagation and increased biomass production (Etesami 2018). Rajkumar et al. (2013) showed an increase in phytostabilization potential for *Brassica juncea*, *Luffa cylindrica* and *Sorgo halepense* plants inoculated with the Ni resistant *Bacillus megaterium* SR28C isolate. The bacteria alleviated the toxicity of Ni by reducing its absorption and translocation in plants. Similarly, Srivastava and Singh (2014) used bacteria immobilizing metal—*Acinetobacter* sp. isolated from arsenic-contaminated soil—to improve plant growth and reduce heavy metal translocation to plant shoots, thus enhancing the potential for phytostabilization of *Cicer arietinum* grown on soils contaminated with arsenic. Moreover, research presented by Ma et al. (2015) using *Psychrobacter* sp. SRS8 and *Pseudomonas* sp. A3R3 bacteria isolated from serpentine soil revealed a significant effect on plant growth as well as translocation and accumulation of Ni, Zn, and Fe by *Brassica juncea* and *Ricinus communis* grown on metal-contaminated serpentine soil. Plant inoculation with bacteria significantly increased plant biomass and heavy metal accumulation compared with the unvaccinated control, which the authors attributed to bacterial production of metabolites that stimulate plant growth and/or mobilize metals. The *Psychrobacter* SRS8 strain showed the maximum increase in biomass of the tested plants, while *Pseudomonas* A3R3 displayed the maximum effect on heavy metal accumulation in both plants. However, both plant species showed low values of the bioconcentration factor (< 1) for Ni and Fe, regardless of inoculation. The authors showed significant increase in the translocation coefficient (TF) for Ni, while the TF value for Zn was reduced in both inoculated plant species.

Plant growth–promoting rhizobacteria (PGPR) were initially used in agriculture and forestry to increase productivity and disease resistance and to protect against stress associated with the presence of trace metals or low pH soils, but also due to flooding, organic toxic substances, high salinity, drought, and phytopathogens (Saleem et al. 2007; Glick 2010; Bhattacharyya and Jha 2012). PGPR influence plants by, e.g., increasing the pool of bioavailable phosphorus, nitrogen, and iron (with siderophore secretion) and producing plant hormones (gibberellins, cytokinins, auxins) (Ma et al. 2015, 2016). They also increase plant resistance, e.g., by decreasing ethylene level (through the synthesis of ACC deaminase) (Saleem et al. 2007; Sessitsch et al. 2013; Goswami et al. 2016). The PGPR include, among others, strains of *Pseudomonas putida*, *Pseudomonas aeruginosa*, *Azospirillum brasilense*, *Serratia liquefaciens*, and *Enterobacter cloacae* (Bhattacharyya and Jha 2012). As He et al. (2009, 2013) showed, the presence of endophytes can significantly affect the efficiency of phytoextraction. The authors (He et al. 2009) studied the effect of two cadmium-resistant strains *Pseudomonas* sp. RJ10 and the *Bacillus* sp. RJ16 on increasing the mobility of cadmium and lead in soil and promoting plant growth Cd and Pb uptake by a tomato cultivar with features of Cd hyperaccumulator. They observed an increase in available forms of Cd and Pb in inoculated soil, by 58–104% and 67–93%, respectively, compared with unvaccinated controls. In the studied tomato plants, the increase in the content of Cd and Pb in aboveground ranged from 70 to over 110%, respectively, in vaccinated plants growing in soil contaminated with heavy metals compared with non-inoculated plants. Inoculation with PGPR also has the potential to increase the efficiency of phytoremediation (He et al. 2013). The authors showed that inoculation of *Brassica napus* plants with *Rahnella* sp. JN6 alleviated the stress caused by the presence of metals due to ACC deaminase secreted by bacteria, and at the same time plants displayed increased root and shoot length and root biomass. Rape plants inoculated with the isolate JN6 had significantly higher concentrations and uptake of Cd, Pb, and Zn in both aboveground and root tissues than those without inoculation grown in soils amended with Cd, Pb, or Zn. These results show that the bacteria can be used to improve bacterial phytoextraction of soils contaminated with Cd and Pb. However, the optimization of parameters for inoculation of selected plants with microorganisms is difficult, the reason being that the influence of bacterial consortium depends on the inoculum density and plant species, as well as on the plant’s stage of development (Karami and Shamsuddin 2010).

Plants’ ability to accumulate metals is expressed normally as an average content of trace elements in grams of dry matter. A large dry biomass production per hectare is critical for soil remediation (Neugschwandtner et al. 2008). Under normal conditions, crop yields can be significantly improved by simultaneous intercropping of two different species through the efficient use of water, nutrients, and solar energy, compared with monoculture cropping (Mead and Willey 1980; Olowe and Adeyemo 2009; Temperton et al. 2007). The so-called companion planting (co-planting) reduces losses caused by diseases and parasites (Held et al. 2003; Wang et al. 2010; Liu et al. 2011). Crop co-planting may affect phytoextraction of metals from soil because coexistence of multiple plant species may change rhizosphere microorganisms, soil enzyme activities, and the abiotic micro-environment, and thus may affect the metal bioavailability in rhizosphere soil (Khan 2005; Yang et al. 2009).

The use of crops in a co-planting system for the phytoextraction of metals has been studied for about 10 years. However, the aim of co-planting was mainly to increase phytoextraction efficiency of hyperaccumulators and metal-accumulating plants by improving their physiological state. Experiments have shown that some plant species can intensively export H^+^ ions and/or exude low molecular weight organic acids (e.g., acetic, oxalic, fumaric, citric, and tartaric acids) into soil, which can increase metal mobility either directly or indirectly by affecting microbial activity (Chiang et al. 2006; Evangelou et al. 2006; Duarte et al. 2007). Moreover, H^+^ can replace cations and make metal cations more bioavailable (Marques et al. 2009). For example, the hyperaccumulator *Sedum alfredii* was cultivated with a low-accumulating variety of *Zea mays* (Wu et al. 2007), or an accumulating variety of *Nicotiana tabacum* with non-accumulating *Kummerowia striata* (Liu et al. 2011). The design to match species and varieties with different abilities to accumulate metals is based on a specific phenomenon: although co-planting physically reduces density and biomass of an accumulating plant, by incorporating a second species, the resulting yield of trace metals in the harvest can be similar to that from a monoculture (Jiang et al. 2010). Another approach involves co-planting to increase the yield of the crop grown on contaminated soil while maintaining a low accumulation of metals in the collected material (Yang et al. 2012).

The aim of this study was to improve the efficiency of phytoextraction of trace elements (zinc, lead, and cadmium) by combining assisted phytoextraction and a co-planting culture. In the course of the pot experiment, *B*. *juncea* was grown individually, with *Zea mays* or with *Medicago sativa*. Half of the pots were inoculated with a plant growth–promoting rhizobacteria (PGPR) inoculation, *Burkholderia phytofirmans* PsJN^T^.

## Material and methods

### Soil description

Around 300 kg of surface soil was collected (0 to 20 cm depth) from a site situated between the towns of Bytom and Piekary Śląskie, in the Upper Silesia Industrial Region of southern Poland. This site is located in proximity to a former mine and smelter area, and was used for agricultural purposes until the early 1980s, when farming ceased due to poor crop yield. The mine and smelter operated for approximately 70 years, and the primary minerals of concern were zinc, lead, cadmium, ore, dolomite, silt, and gravel. The metal ores were thermally processed on-site, applying the Welz and Doerschel process (Stuczyński et al. 2000). Mining activities resulted in land deformations, subsidence, and a considerable lowering of the groundwater table. In 1989, production stopped, all the facilities were closed down and dismantled, and the revitalization of the area (460 ha) was attempted. Many of the old tailing piles and surrounding wastelands are overgrown with grasses and short trees, although a large area remains unvegetated (Kucharski et al. 2005). Garden soil (ecological universal soil, pH 5.5–6.5, obtained from a local distributor) was used to dilute the contaminated soil collected from Piekary Śląskie. Soil was stored at room temperature, thoroughly mixed in the appropriate proportions (1:1 and 1:3), sieved (3 mm), and used for further experiments.

### Physicochemical soil parameters

Soil pH was measured in deionized water (1:2.5 m/v) and 1 M KCl (1:2.5 m/v) with a combination glass/calomel electrode and a pH/conductivity meter (CPC-505, Elmetron, Poland) at room temperature after 24 h of equilibration. The electrical conductivity (EC) was determined in deionized water suspension (soil-to-solution ratio 1:2.5 m/v) at room temperature after 24 h of equilibration by using a glass conductivity cell (EC-60, Elmetron, Poland) and a pH/conductivity meter (CPC-505, Elmetron, Poland). The content of bioavailable forms of metals was obtained using extraction with 0.01 M CaCl_2_. Extraction was conducted with 3 g of soil (< 2.0 mm) and 30 mL 0.01 M CaCl_2_ for 2 h. The total metal content was determined after digestion of soil ground to < 0.25 mm by using microwave mineralization (ETHOS 1, Milestone, Italy) according to the procedure provided by the manufacturer (concentrated HNO_3_ and H_2_O_2_, 4:1 v/v). The concentration of metals was analyzed in the extracts and digests by using flame atomic absorption spectrophotometry (iCE 3500 FAAS, Thermo Scientific, USA). The reference soil material (NCS DC 77302, China National Analysis Center for Iron and Steel, Beijing, China) was used for quality assurance of analytical data.

### Germination tests

The following plant seeds were used: *Brassica juncea* (L.) Czern. “Małopolska,” *Medicago sativa* L. “Sanditi” (Barenbrug, Poland), *Zea mays* L. “Codimon” C1 INFLUX XL (Oseva, Poland). Bacteria *Burkholderia phytofirmans* PsJN^T^ (the strain was kindly provided by prof. Angela Sessitsch from the Austrian Institute of Technology GmbH) were grown in TSB liquid media (Merck) until the exponential growth phase, as measured by OD_600_. Germination tests were carried out using PhytoToxKit plates (Tigret, Poland), according to the manufacturer’s instruction. A buffer (30 mL) containing 1.48 g Na_2_HPO_4_ × 12 H_2_O, 0.28 g KH_2_PO_4_, 0.05 g NaCl, and 0.1 g NH_4_Cl suspended in 1 L of sterile water was mixed with 85 g of garden soil or garden soil mixed in 1:1 or 1:3 w/w proportions with contaminated soil. The preliminary tests showed that the growth of the crop plants (*Zea mays*, *Brassica juncea*, and *Medicago sativa*) was heavily inhibited on contaminated soil collected from Piekary Śląskie. Because germination tests showed a strong negative effect of the 1:3 mixture of soil (3 parts by weight of soil from Piekary Śląskie and 1 part by weight of garden soil), especially on the growth and development of *B*. *juncea*, it was decided that long-term pot cultivation would be conducted on a 1:1 mixture. Then, 10 or 7 seeds of *B*. *juncea*, *M*. *sativa*, or *Z*. *mays* were sowed on each pot, respectively. The choice of inoculum density was based on previous studies of this strain (Compant et al. 2008). It was decided to assess the influence of using the inoculum at four densities: 7.06 × 108, 7.06 × 10^8^, 1.41 × 10^9^, 2.82 × 10^9^, 5.65 × 10^9^ (CFU kg^−1^ of soil). Inoculum density was selected for further studies, which showed the lowest negative impact on germination of three species in this experimental system, 1.41 × 109 CFU kg^−1^ soil. Non-inoculated buffer was used for the control plates. To minimize the level of stress at the early stage of plant development (simultaneous abiotic stress due to the presence of metals and biotic due to bacterial colonization), plant inoculation was carried out 7 days after sowing. The germination tests were performed in triplicates.

### Greenhouse pot experiments

The pot culture was carried out in an automated greenhouse at the Greater Poland Center for Advanced Technologies (Poznań, Poland). Growing conditions: temperature between 6:00 a.m.–22:00 p.m.–21.5–22.5 °C, 22:30 p.m.–5:30 a.m.– 18–19.5 °C; humidity: 35–40%; complementary lighting: from 6:00 a.m. to 22:00 p.m. to 100 Wm^−2^. Seeds were sown in 1-L pots. Plant seeds were inserted into the pots to a depth of 0.5 cm: 12 seeds of *B*. *juncea*, 6 seeds of *B*. *juncea* + 2 seeds of *Z*. *mays*, 6 seeds of *B*. *juncea* + 10 seeds of *M*. *sativa*. After 2 weeks of cultivation, the number of plants was limited by half in pots by cutting the shoot near the ground. Ultimately, the experimental setup consisted of 3 cultivation variants conducted independently for control plants and inoculated with PGPR bacteria: 6 pots with only *B*. *juncea* plants (6 plants in each), 3 pots of *B*. *juncea* (3 plants in each) plus of *Z*. *mays* (1 plant in each), and 3 pots of *B*. *juncea* (3 plants in each) plus *M*. *sativa* (5 plants in each). The plants were watered three times a week using a mixture with Florovit Universal liquid fertilizer (INCO Group, Poland) at 5 mL per liter of distilled water. After a week, plants were inoculated with *B*. *phytofirmans* suspended in 30 mL buffer described in the “Germination tests” section, using an inoculum density of 1.41 × 109 CFU kg^−1^ soil. Uninfected buffer was used in control pots. Inoculated and non-inoculated plants were grown in separate flooding tables. Cultivation was carried out for 6 weeks from sowing to harvest. As part of each experimental series, each variant was represented by three pots, prepared and treated in the same way. The described pot experiment was carried out three times in 4 months (from May to August).

### Sample preparation

Plant material (roots, stems, and leaves) was rinsed with distilled water, gently dried on blotting paper, weighed, and dried at 70 ± 2 °C. The dried samples were mineralized in a microwave digestion oven (Ethos One, Milestone, Italy). The samples for digestion were prepared as follows: approximately 0.5 g of the sample was transferred to digestion vessels and 5 mL of 65% nitric acid (Merck, Germany) was added to each vessel. The microwave oven heating program proceeded in steps: (1) ramp time of 20 min to reach 1500 W, (2) hold time of 30 min at 1500 W, and (3) cooling for 30 min. The temperature during the digestion process was 220 °C. After mineralization, samples were quantitatively transferred to 10-mL flasks and filled with deionized water. In parallel, the procedural blanks, including the same reagents as the samples, were prepared and digested in the same way as the samples in each digestion run.

### Analytical procedure

An inductively coupled plasma mass spectrometry (ICP-MS) model Elan DRC II (Perkin-Elmer Sciex, Canada) was used to determine the concentration of Cd, Cu, Pb, and Zn in the mineralized plant tissues. An ICP-MS spectrometer equipped with a Meinhard concentric nebulizer, cyclonic spray chamber, Pt cones, and quadrupole mass analyzer was used for this study. Argon with a purity of 99.999% was used as a nebulizer, auxiliary, and plasma gas (Linde Gaz, Poland). As the DRC reaction gas, high-purity ammonia (99.999%) was used. Deionized water was used throughout the experiment. Treated and control plant materials were analyzed ex vivo by an LA-ICP-MS. The ICP-MS spectrometer model Elan DRC II (Perkin-Elmer Sciex, Canada) was equipped with an Nd:YAG laser ablation system (LSX-500, CETAC Technologies, Omaha, NE, USA) operating at a wavelength of 266 nm. The accuracy of the results obtained with the LA-ICP-MS method depends on the following: distribution of the analyzed on a sample’s surface, homogeneity of the matrix, and geometry of the sample (Hanć et al. 2016). The exact description for the ICP-MS and LA-ICP-MS parameter optimization has been described in Supplementary Table 1.

### Analytical performance

After calibration, and also during the analysis, measurements were controlled by analysis of standard solutions at concentrations of 1 μg L^−1^ or 5 μg L^−1^ and certified reference materials after each batch of fifteen samples. The calibration curves for the determined elements were linear in the range of calibration standards. The correlation coefficient *R* exceeded a value of 0.999. The trueness of the analytical results was assessed using the reference material NIST SRM 1515 Apple Leaves and NIST SRM Spinach Leaves 1575a. The accuracy of the method for the investigated elements was evaluated by determining the percentage bias between the measured concentration of the applied certified reference materials (CRMs) and its certified value. The bias represents the difference between the CRM elemental concentration measured using ICP-MS and the certified value, which is as follow: 1.5% for Cd, 2.3% for Cu, 1.7% for Pb, and 2.5% for Zn. The limits of detection (LOD) for the determined elements were counted according to LOD = 3.3 *S*/*b*, where *S* means standard deviation of the result obtained for the blank samples and *b* is the sensitivity. The LODs for the ICP-MS method were found to be 0.02 μg g^−1^(Cd), 0.05 μg g^−1^ (Cu), 0.008 μg g^−1^ (Pb), and 0.01 mg g^−1^ (Zn). LOQ values were calculated as three times the LOD values. Precision was calculated as the relative standard deviation expressed as %. As a result of the analysis, the precision values were calculated for Cd (1.2%), Cu (2.8%), Pb (1.7%), and Zn (2.4%).

### Chlorophyll content measurement

The level of chlorophyll *a* and *b* was estimated using DMSO according to the method described by Ronen and Galun (1984). Leaves (200 mg) from *B*. *juncea* plants were cut into small (4–16 mm^2^) pieces and placed in a vial with 5 mL DMSO. Three replicates of samples were incubated in a water bath at 65 °C for 120 min. Chlorophyll extract was transferred to a cuvette and spectrophotometric readings were made at 649 nm and 665 nm using a UV–VIS spectrophotometer (Shimadzu Scientific Instruments, Japan).

### Measurements of the level of reactive oxygen species

Reactive oxygen species (ROS) levels were determined in *B*. *juncea* shoots grown with *Z*. *mays* and *M*. *sativa* plants, inoculated and non-inoculated with PGPR. Superoxide anion content was determined according to Doke (1983) at 580 nm. The plant shoots (0.5 g) were placed in test tubes and filled with 7 mL of mixture containing 50 mM phosphate buffer (pH 7.8), 0.05% NBT (nitro blue tetrazolium) and 10 mM of NaN_3_. Next, the test tubes were incubated in darkness for 5 min, after which and then 2 mL of the solution was taken from the tubes heated at 85 °C for 10–15 min and cooled in ice for 5 min. The absorbance was measured using spectrophotometry (SHIMADZU UV-1800, Japan) at 580 nm against the control.

Hydrogen peroxide content was determined according to Patterson et al. (1984). The plant shoots were homogenized in 5% TCA (trichloroacetic acid). The homogenate was centrifuged twice at 13,000*g* for 20 min. The level of hydrogen peroxide was determined in the supernatant by the spectrophotometric method at 508 nm. The reaction mixture contained 50 mM phosphate buffer (pH 8.4), a reagent containing 0.6 mM 4-(-2 pyridylazo) resorcinol, 0.6 mM potassium-titanium oxalate in 1:1. A corresponding concentration of H_2_O_2_ was determined against the standard curve of H_2_O_2_.

### Determination of antioxidative enzyme activities

Plant shoots (0.5 g) were homogenized in isolation buffer 50 mM K_2_HPO_3_/KH_2_PO_4_, pH 7.0; 1% Triton X-100; l7 mM 2-mercaptoethanol, and 1 mM ascorbic acid at 4 °C. The homogenate was centrifuged twice at 13,000*g* for 20 min. The supernatant activity of antioxidant enzymes was determined. Activity of SOD was assayed according to Beauchamp and Fridovich (1971), with slight modification. The activity was assayed by measuring its ability to inhibit the photochemical reduction of NBT. The reaction mixture contained 13 mM riboflavin, 13 mM methionine, 63 mM NBT, and 50 mM potassium phosphate buffer (pH 7.8). Absorbance at 560 nm was then measured. One unit of SOD activity was defined as the amount of enzyme, which causes a 50% decrease of the inhibition of NBT reduction. Activity of CAT was determined according to Aebi (1983) at 240 nm. The activity of CAT was determined by directly measuring the decomposition of H_2_O_2_ at 240 nm for 3 min in 50 mM phosphate buffer (pH 7.0) containing 5 mM H_2_O_2_ and enzyme extract. CAT activity was determined using an extinction coefficient of 36 mM^−1^ cm^−1^ for H_2_O_2_. Activity of APOX was determined according to Nakano and Asada (1981). The method relies on monitoring the rate of ascorbate oxidation at 290 nm (extinction coefficient of 2.9 mM^−1^ cm^−1^) for 3 min. The reaction mixture consisted of 25–50 μL supernatant, 50 mM phosphate buffer (pH 7.0), 10 mM H_2_O_2_, 0.2 mM ascorbate, and 0.2 mM EDTA.

### Protein quantification

Total soluble protein contents were determined according to Bradford (1976), using the BioRad assay kit with bovine serum albumin as a calibration standard.

### Dehydrogenase activity in soil

Measurement of dehydrogenase activity by microorganisms in soil has the potential to serve as a useful indicator of microbial activity. Soil dehydrogenase activity was measured by the reduction of 2,3,5-triphenyl tetrazolium chloride (TTC) to 1,3,5-triphenyl formazan (TPF) with the Penrose and Glick method (Penrose and Glick 2003). A soil sample (2.5 g) was incubated for 24 h at 23 °C in 5 mL of 1% TTC solution. After incubation, the sample was blended with 10 mL of methanol to extract TPF and shaken for 1 min, then filtered. Absorbance in the extract was measured at 485 nm. Finally, soil dehydrogenase activity was calculated as μg TPF g^−1^ dry soild^−1^.

### Western blot and immunodetection of CuZnSOD and FeSOD

Western blot analysis was performed for protein extracts from shoot seedlings of *B*. *juncea*, grown in a monoculture and in co-planting culture with *M*. *sativa* and *Z*. *mays*, in the presence and non-presence PGPR. RIPA buffer (150 mM NaCl, 1% Triton X-100, 0.5% Na deoxycholate, 0.1% SDS, 50 mM Tris, pH 8.0) was used to lyse the cells. The protein concentrations were determined using the Bradford method and 50 μg of each fraction was loaded on the gel. Proteins were separated on a 12% resolving SDS-PAGE gel. Immunodetection was carried out using primary polyclonal antibodies raised against CuZnSOD (chloroplastic Cu/Zn superoxide dismutase) or FeSOD (chloroplastic Fe superoxide dismutase) (Agrisera antibodies) at a dilution of 1:1000 and goat anti-rabbit horseradish peroxidase-conjugated secondary antibodies (BioRad) at a dilution of 1: 50000. CuZnSOD and FeSOD bands were visualized using the Amersham ECL system and quantified digitally using the Scan Pack 3.0 program. The results are presented as the mean ± S.E. obtained from 2 independent experiments (plant growths and preparations), and each determination was performed at least in triplicate throughout the study.

### Statistical analysis

Experiments were carried out in three biological and technical repetitions. Average values (± SD, standard deviation) are given in tables and diagrams. The results were analyzed using the IBM SPSS Statistics program (Version 22 for Windows). Statistically significant differences between the variants were analyzed using the one-way ANOVA method, at *p* < 0.05, and using the post hoc b-Tukey test. If no letters are marked on the charts, it means that either the b-Tukey test did not show a statistically significant difference or it was impossible to compare these variants due to the too low number of independent measurements. For experiments using germination tests and pot culture, box plots were used to show the distribution of the characteristics of the analyzed samples, in the case of collecting *n* ≥ 5 samples for a given variant. In other cases, the data are presented as mean values (± SD). Box plots have been constructed as follows: the top and bottom sides of the rectangle are equal to Q3 and Q1 quartiles, a median is marked in the middle of the rectangle, the width of the box corresponds to the value of the interquartile range (IQR), i.e., the difference between the third and the first quartiles, whiskers (upper and lower) show the range of the highest and lowest measurements lying within 1.5*IQR, single points are measurements outside the range of 1.5*IQR (outside internal limits).

## Results

The parameters of soil used in the course of experiments are presented in Table 1. Soil collected from Piekary Śląskie with garden soil in the mixture of 1:1 had a pH of 6.90 and was enriched in Cd (22.46 mg kg^−1^ DW), Pb (615 mg kg^−1^ DW), and Zn (1822 mg kg^−1^ DW). The results indicate that the level of the total metal content for the three elements in the soil was exceeded: zinc (sixfold), lead (sixfold), and cadmium (fivefold).

**Table 1:** Properties of soil used in cultivation of prepared mixture (1:1) from garden soil and soil collected from Piekary Śląskie

MIXTURE of soil in pots (1:1; garden soil and soil from Piekary Śląskie)					
	Total content (mg kg^−1^ of DW)	Bioavailable metal content (mg kg^−1^ of DW)	pH_H2O_	pH_1M KCl_	EC (μS cm^−1^)
Cd Cu Fe Mg Mn Pb Zn	22.46 ± 1.76 17.19 ± 1.09 10,573 ± 903 1965 ± 15 484 ± 26 615 ± 36 1822 ± 166	0.696 ± 0.023 0.295 ± 0.069 5.75 ± 1.82 177.3 ± 3.1 25.24 ± 0.78 2.52 ± 0.79 54.1 ± 6.9	6.90	6.80	1203.89

**Fig. 1:**
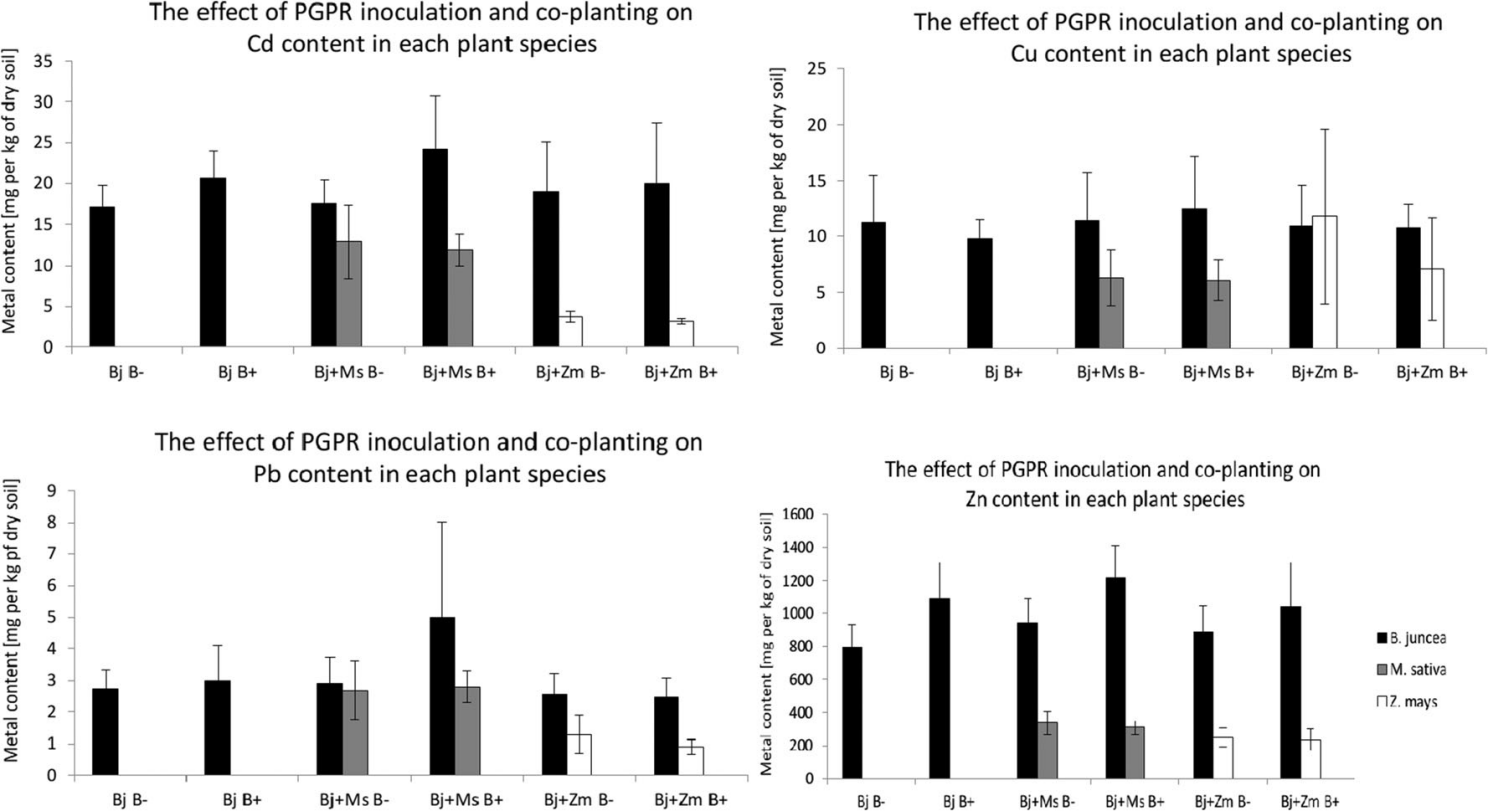
Influence of inoculation of *Burkholderia phytofirmans* and co-planting cultivation (Bj + Zm; Bj + Ms) on the metal content (Cu, Cd, Pb, Zn) in shoots of plants *B*. *juncea*, *M*. *sativa*, and *Z*. *mays* grown in pots with garden soil and from Piekary Śląskie (MIXTURE 1:1) in variants: Bj B−, Bj B+, Bj + Ms B−, Bj + Ms B+, Bj + Zm B−, Bj + Zm B+. Bj - *B*. *juncea*, Ms - *M*. *sativa*, Zm - *Z*. *mays*, “B−” - without bacterial inoculation, “B+” - inoculated plants. Mean values of three replicates (± SD)

The average content of metals (Cu, Cd, Zn, Pb) in *B*. *juncea* shoots was higher by about fivefold than their content in *Z*. *mays* and *M*. *sativa* with the exception of Pb content in *M*. *sativa* and Cu in *Z*. *mays* (Fig. 1). Microbial inoculation generally increased metal content in *B*. *juncea.* There was no statistically significant impact of companion planting cultivation on the content of metals in *B*. *juncea* plants. The highest accumulation was observed for Zn and it was about 50 to 240 times higher in *B*. *juncea* shoots than other elements, while the lowest accumulation was found for Pb.

**Fig. 2:**
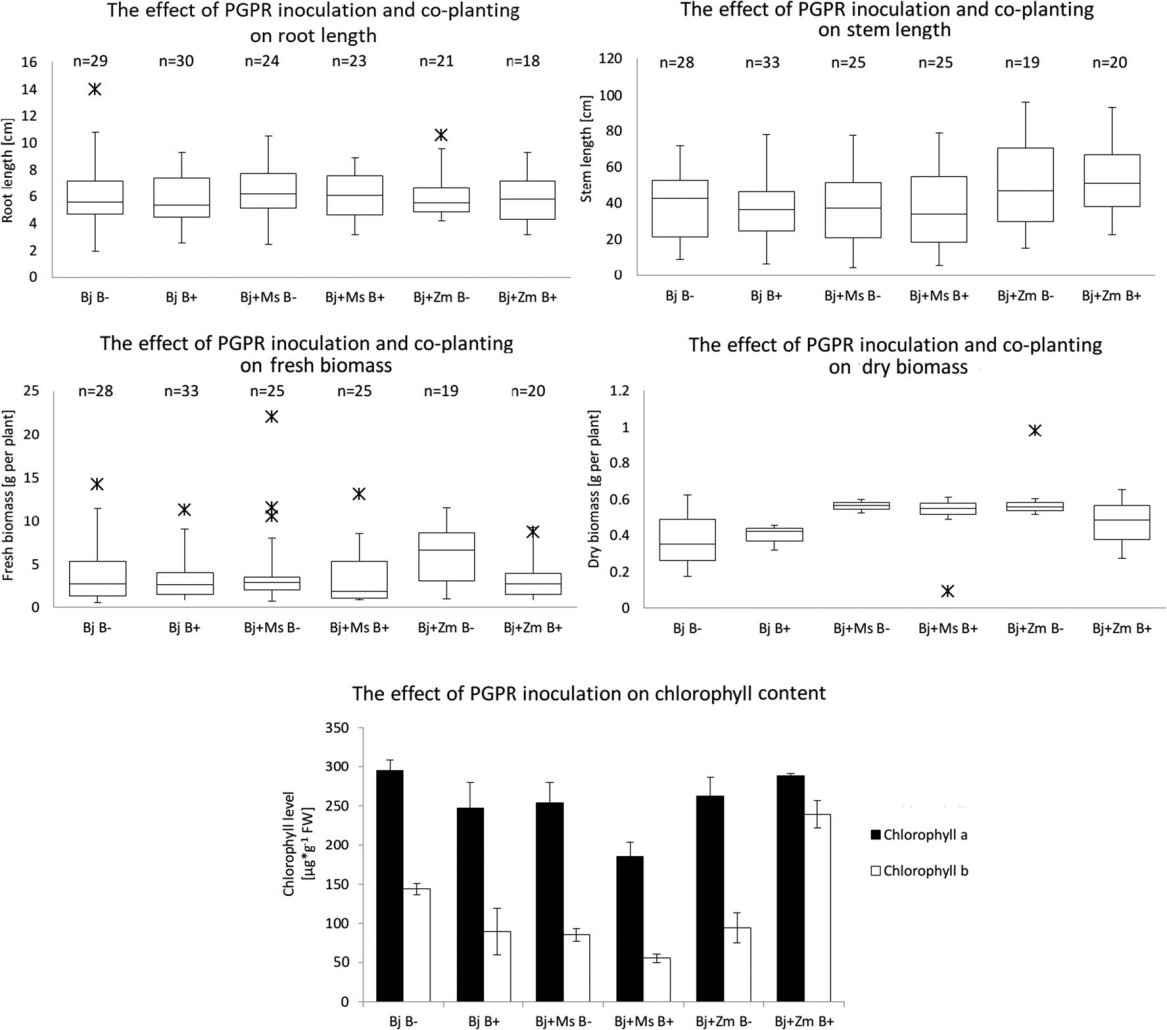
Effect of *Burkholderia phytofirmans* inoculation and co-planting cultivation on plant growth parameters (root and stem length; fresh and dry biomass of cuttings) and chlorophyll content in the leaves of *B*. *juncea*, *M*. *sativa*, and *Z*. *mays*. Plants grown in pots with garden soil and from Piekary Śląskie (MIXTURE 1: 1) in variants: Bj B−, Bj B +, Bj + Ms B−, Bj + Ms B+, Bj + Zm B−, Bj + Zm B+. Bj - *B*. *juncea*, Ms - *M*. *sativa*, Zm – *Z*. *mays*, “B−” - without bacterial inoculation, “B+” - inoculated plants. Mean values of three replicates (± SD)

No significant differences were observed in the root length of the plants inoculated with PGPR and under the influence of co-planted culture (Fig. 2). In the case of stems, the most positive result was observed for variant *B*. *juncea* with co-planting with *Z*. *mays*, both inoculated and non-inoculated bacteria. Co-planting culture of *B*. *juncea* and *Z*. *mays* plants had the greatest impact on the fresh mass, whereas in the other variants, no significant differences were observed. Inoculation with PGPR bacteria did not increase fresh weight in the tested plants. The greatest effects of coordinate cultivation and inoculation with the *Burkholderia phytofirmans* PsJN^T^ strain can be seen when measuring the dry weight of plant seedlings. The dry mass of seedlings in variants *B*. *juncea* with *Z*. *mays* and *B*. *juncea* with *M*. *sativa*, both with and without bacteria, was on average 1.5-fold higher compared with control plants. The content of chlorophyll *a* and *b* increased significantly in only one research variant: *B*. *juncea* with *Z*. *mays* inoculated with PGPR. In other variants, a decrease in chlorophyll content was observed in the case of bacterial inoculation.

**Fig. 3:**
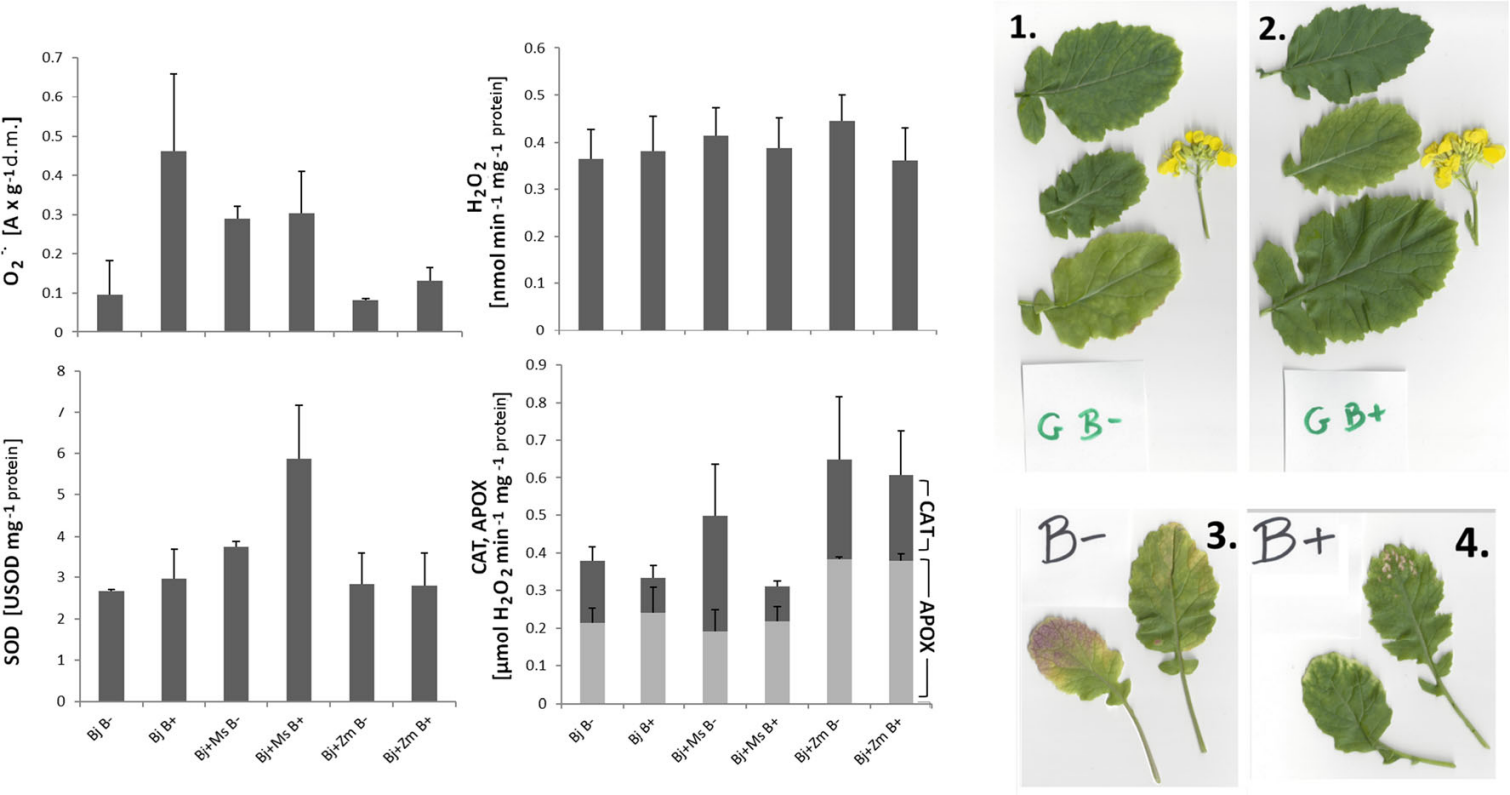
Left panel: Influence of *Burkholderia phytofirmans* inoculation and co-planting cultivation on the level of ROS (hydrogen peroxide and superoxide anion) and SOD CAT, APOX activities in *B*. *juncea* shoots grown in pots with garden soil and from Piekary Śląskie (MIXTURE 1: 1) in variants: Bj B−, Bj B+, Bj + Ms B−, Bj + Ms B+, Bj + Zm B−, Bj + Zm B+. Bj - *B*. *juncea*, Ms - *M*. *sativa*, Zm – *Z*. *mays*, “B−” - without bacterial inoculation, “B+” - inoculated plants, APOX - ascorbate peroxidase, CAT - catalase, SOD - superoxide dismutase. Right panel: Influence of *Burkholderia phytofirmans* inoculation on *B*. *juncea* shoot plants. Representative leaves and flowers of *B*. *juncea* from the control group without inoculation (1) and after PGPR inoculation (2). Most frequently observed changes on the leaves: for control plants - violet coloration (3), for inoculated plants - chlorosis (4). Mean values of three replicates (± SD)

In most research variants, an increase in the level of ROS was observed in response to both biotic and abiotic stress factors (Fig. 3). The superoxide anion level in *B*. *juncea* was increased for PGPR inoculation variants (“Bj B+,” “Bj + Zm B+,” “Bj + Ms B+”), compared with the corresponding non-inoculation variants (“Bj B−,” “ Bj + Zm B−,” “ Bj + Ms B–”) on average from 1 to 4 times. At the same time, a reduction in the hydrogen peroxide level and CAT activity was observed in variants after inoculation with PGPR (except for “Bj B+”). In plants inoculated with PGPR (Fig. 3), an increased level of O_2_^•−^ and SOD activity as well as reduced CAT activity was observed compared with the control plants for each cultivation variant, except for the variant of simultaneous cultivation of *B*. *juncea* and *Z*. *mays* (“Bj + Zm”). There were no significant differences in the activity of the third important antioxidant enzyme—APOX—in either inoculated or control plants. In addition, “Bj + Zm” was the variant from which the smallest number of *B*. *juncea* plants was harvested after cultivation, suggesting a high level of oxidative stress. We observed the effect of the *Burkholderia phytofirmans* PsJN^T^ strain on morphological changes of *B*. *juncea* leaves and flowers. We noticed the positive effect of PGPR bacteria on plant development. The violet coloration of the leaves was a frequent symptom of stress, characteristic of plants without inoculation. In the case of inoculated plants, violet coloration of the leaves was only rarely observed. The most common symptom of stress in this group of plants was chlorosis.

The level of CuZnSOD protein was decreased in *B*. *juncea* plants inoculated with *phytofirmans* PsJN^T^ strain, in comparison with non-inoculated plants, in variants grown in monoculture and co-planted with *M*. *sativa*. Regarding the FeSOD level, the differences were not statistically significant (Fig. 3).

We noticed that inoculation with the PGPR bacteria *B*. *phytofirmans* PsJN^T^ strain led to an increase in phytoextraction efficiency in most cases (Table 2). The highest negative effect of inoculation was observed for the yield of *B*. *juncea* plants co-planted with *Z*. *mays* for Cu and Pb metals. However, the total hypothetical metal yield for this variant (sum of *B*. *juncea* and *Z*. *mays* yield) showed an increase in phytoextraction efficiency for Zn, while for Cu and Pb, no significant differences were observed. The highest efficiency of phytoextraction was obtained in the variant of the *B*. *juncea* co-planted with *M*. *sativa* combined with PGPR inoculation—an increase of 95% for Zn, 90% for Cd, and approx. 160% for Pb.

**Table 2:** The influence of *Burkholderia phytofirmans* inoculation and companion planting cultivation on the efficiency of trace metal phytoextraction (Zn, Cd, Pb). It was calculated on the basis of the recorded cultivation parameters: the number of collected plants, the average dry biomass of the plants, and the average metal content in all plant from gatunek (yield). The total yield is the sum of the calculated yield of all cultivated plants for a given variant (i.e., the sum of the *B*. *juncea* yield “Bj + Ms B−” and *M*. *sativa* “Ms B−” from the co-planting variant)

Variant of planting cultivation [$\overline{x\ }$±SD]											
		Bj B−	Bj B+	Bj + Ms B−	Bj + Ms B+	Bj + Zm B−	Bj + Zm B+	Ms B−	Ms B+	Zm B−	Zm B+
Me content [mg g^−1^]	Cd	0.022 ± 0.008	0.023 ± 0.006	0.021 ± 0.006	0.022 ± 0.007	0.022 ± 0.006	0.021 ± 0.005	0.013*	0.012*	<LOD	<LOD
	Pb	0.003*	0.003*	0.003*	0.005*	0.003*	<LOD	0.003*	0.003*	<LOD	<LOD
	Zn	0.790 ± 0.032	1.090 ± 0.044	0.941 ± 0.042	1.211 ± 0.046	0.892 ± 0.038	1.042 ± 0.051	0.340 ± 0.027	0.311 ± 0.031	0.250 ± 0.021	0.243 ± 0.022
Yield [mg]	Cd	0.212 ± 0.012	0.305 ± 0.026	0.252 ± 0.013	0.33 ± 0.016	0.201 ± 0.012	0.193 ± 0.011	0.042 ± 0.002	0.071 ± 0.004	0.092 ± 0.005	0.092 ± 0.005
	Pb	0.033 ± 0.027	0.042 ± 0.032	0.042 ± 0.005	0.071 ± 0.004	0.032 ± 0.002	0.022 ± 0.002	0.013 ± 0.003	0.022 ± 0.002	0.031 ± 0.002	0.031 ± 0.002
	Zn	9.47 ± 0.416	15.61 ± 0.672	13.27 ± 0.66	16.63 ± 0.83	9.45 ± 0.47	9.74 ± 0.48	1.03 ± 0.051	1.78 ± 0.092	6.10 ± 0.31	7.09 ± 0.36
Total yield [mg]	Cd	0.210 ± 0.011	0.301 ± 0.015	0.291 ± 0.015	0.401 ± 0.021	0.291 ± 0.015	0.282 ± 0.013				
	Pb	0.032 ± 0.002	0.041 ± 0.002	0.052 ± 0.003	0.084 ± 0.004	0.063 ± 0.003	0.052 ± 0.003				
	Zn	9.47 ± 0.46	15.61 ± 0.78	14.30 ± 0.72	18.41 ± 0.92	15.55 ± 0.82	16.83 ± 0.91				
Plant [n]		31	36	25	25	19	20	53	56	9	9

*Bj*, *B*. *juncea*; *Ms*, *M*. *sativa*; *Zm*, *Z*. *mays*; “*B−*,” without bacterial inoculation; “*B+*,” inoculated plants. Mean values of three replicates (± SD)

*Information mass fraction value (the value below LOD)

**LOD values for Cd, Pb, and Zn were 0.02 μg g^−1^, 0.008 μg g^−1^, and 0.01 μg g^−1^, respectively

## Discussion

### *B*. *juncea*—plant useful in the phytoextraction

In times of increased anthropogenic activity, soil pollution is a serious problem. Several methods are available to remediate soil contaminated with metals, though most of them are expensive and laborious (e.g., excavation of a contaminated material and an off-site treatment). Additionally, soil properties are severely altered after such treatment (Leštan et al. 2008). Phytoextraction is an alternative approach that applies plants for metal removal, either off-site after excavation or on-site. Phytoextraction has become a tangible alternative because it is an environmentally friendly and cost-effective method. There are two strategies for phytoextraction: removal performed by plants with the ability to accumulate high amounts of metals (preferably in the aboveground parts), and removal assisted by plants with a high biomass yield, supplemented with substances to increase the metal uptake (Leštan et al. 2008).

*B*. *juncea* has been chosen as a primary plant for our research because of its ability to accumulate trace metals, as shown in both lab-scale and field-scale experiments (Rascio and Navari-Izzo 2011; Kutrowska et al. 2017). As demonstrated earlier, *B*. *juncea* can accumulate Pb and Cd (Jiang et al. 2000; Meyers et al. 2008) as well as Cr, Cu, Ni, Pb, and Zn (Prasad and de Oliveira Freitas 2003; Babula et al. 2012). It belongs to Brassicaceae, a family rich in metallophytes (among others from the *Noccaea caerulescens*, *Brassica*, *Arabidopsis genera*) (Kramer 2010). Literature analysis of experiments involving *B*. *juncea* shows that this plant is susceptible to the positive influence of microbial inoculation and can be stimulated to increase metal phytoextraction rate. Inoculation with different PGPR can increase metal content in *B*. *juncea* shoots, e.g., up to twofold for copper (Ma et al. 2009) or up to twofold for lead (Wu et al. 2006).

As complementary plants, we chose *Medicago sativa* and *Zea mays plants*; *M*. *sativa* is a Fabaceae plant that in the field enters into symbiosis with rhizobia, which can increase the availability of nitrogen for both their host and its accompanying plants (Markmann and Parniske 2009). There are studies describing the use of *M*. *sativa* for stimulated phytoextraction (e.g., with EDTA) (Lopez et al. 2005), metal rhizofiltration from aqueous solutions (Tiemann et al. 2002), and phytostabilization (Neuman and Schafer 2006). In turn, *Z*. *mays* is one of the most frequently studied species in terms of phytoextraction-supported chelators, due to its rapid biomass growth and high tolerance to stress (e.g., Komarek et al. 2007; Zhao et al. 2010; Niu et al. 2012). In addition, the *Z*. *mays* strategy for the uptake of Fe from the environment is different to that of *B*. *juncea* and *M*. *sativa.* Namely, *Z*. *mays* is able to synthesize phytosiderophores, natural chelators that increase the mobility of metals in soil (Curie et al. 2009; Rajkumar et al. 2010).

### Influence of PGPR on plants

In the presented experiments, we used *B*. *phytofirmans* PsJN^T^ as an inoculum. It is a strain characterized by high activity of ACC deaminase and ability to produce indolylacetic acid which stimulates root growth (Sessitsch et al. 2005; Weilharter et al. 2011). It is known that the impact of PGPR depends on a number of parameters, including plant genotype, inoculum density, and inoculation method (e.g., inoculum temperature) (Pillay and Nowak 1997). It also depends on the stage of the plant development and a plant’s physiological state, because the colonization of plants is associated with the induction of stress (Van Loon 2007). In addition, the effect of a single seed inoculation may also persist at the mature plant stage (Poupin et al. 2013).

Preliminary tests showed a strong inhibition in the growth of the tested plant species (*Zea mays*, *Brassica juncea*, and *Medicago sativa*) on contaminated soil taken from Piekary Śląskie (data not shown). Most likely, contamination with many metals, especially Pb and Zn, contributed to the observed marked effects on germination and plant growth. It was necessary to supplement the soil from Piekary Śląskie with organic compounds by mixing it with garden soil.

Many studies indicate the significant role of bacteria promoting growth in the extraction and removal of trace elements from contaminated soil, among others by increasing biomass growth, which in turn leads to an increase in the efficiency of metal extraction. Examples of microbial-induced promotion of plant growth and increasing stress resistance in phytoextraction studies can be found in crops, hyperaccumulators, and trees. The effect of increasing tolerance on stress is most often associated with the reaction catalyzed by the enzyme ACC deaminase leading to a reduction of ethylene levels in the plant (Arshad et al. 2007; Glick 2003, 2010).

### Effect of PGPR inoculation on the uptake and translocation of metals in plants

The analysis of metal content (Fig. 1) in the studied plant shoots showed a positive effect of PGPR inoculation on the uptake and translocation of Cd, Zn, and Pb in *B*. *juncea* plants, in comparison with non-inoculated plants. However, the inoculation of PGPR did not have any significant effect on the content of metals in *Z. mays and M. sativa* from the co-planted variants with the *B. juncea.* There are studies that show a correlation between higher biomass production with enhanced remediation. Bacteria containing ACC deaminase modulate accelerated production of ethylene in plants treated with metals, and might cause an enhanced uptake of inorganic contaminants through modification of root architecture and also the metal uptake system of the root. *Nicotiana tobacco* plants inoculated with *Pseudomonas putida* UW4 showed an increase in both growth and metal accumulation from nickel-contaminated soil (Li et al. 2007). Similarly, Belimov et al. (2005) reported a positive correlation between ACC deaminase activity of the bacteria and enhanced accumulation of cadmium in *Brassica juncea* tissues through enhanced root growth. The authors suggested that bacteria with ACC deaminase could be used for phytoremediation of metal-contaminated soils. It was found that inoculation with rhizobacterial strains belonging to the genera *Burkholderia*, in both hydroponically and soil-grown plants of *S*. *alfredii*, at Cd/Zn-hyperaccumulator, improved metal tolerance, biomass production, and mostly Cd uptake and extraction (Li et al. 2007; Guo et al. 2010). Moreover, Wu et al. (2006) noted a decrease in cadmium phytotoxicity and an increase in Cd accumulation of up to 40% in a sunflower plant root inoculated with a strain of *Pseudomonas putida* 06909.

### Defensive antioxidative mechanisms in PGPR inoculated and in co-planting plants

Trace metals induce the generation of ROS, including the superoxide radical (O_2_^•−^) and hydrogen peroxide (H_2_O_2_). This can cause cell death due to oxidative stress such as membrane lipid peroxidation, protein oxidation, enzyme inhibition, and damage to nucleic acids. To repair the metal-induced negative effects of ROS, plants employ antioxidant defense mechanisms. Among antioxidative enzymes, superoxide dismutase (SOD; EC, 1.15.1.1) constitutes the primary step of cellular defense and dismutates O_2_^•−^ to H_2_O_2_ and O_2_. Further, the accumulation of H_2_O_2_ is converted to H_2_O through the action of catalase (CAT; EC, 1.11.1.6) or ascorbate peroxidase (APX; EC, 1.11.1.11). Increased levels of superoxide anions and SOD activity, observed in the vaccinated plants, should result in a dismutation reaction to increased production of hydrogen peroxide. However, in the same plants (Fig. 3), small differences (statistically insignificant) in the level of hydrogen peroxide and a decrease in the level of CAT activity were observed (with the exception of the “Bj + Zm B+” variant). This may suggest the participation of other hydrogen peroxide decomposing enzymes (e.g., other peroxidases) in response to stress (Neill et al. 2002; Slesak et al. 2007). Kohler et al. (2009) also observed a decrease in CAT activity (by 55%) under the influence of PGPR inoculation with *Pseudomonas mendocina*. A similar decrease in CAT activity was observed by Upadhyay et al. (2012) in wheat inoculated with *Bacillus subtilis* and *Arthrobacter* sp. and also by Sandhya et al. (2010) in maize inoculated with *Pseudomonas* sp. cultivated under salt stress conditions. In addition, Kohler et al. (2009) observed increased total peroxidase activity in lettuce under the influence of salt stress and inoculation with arbuscular fungi. The change in plant response to biotic stress (presence of PGPB), not only abiotic (presence of heavy metals), is also confirmed by a reduction in the frequency of the appearance of a violet color of leaves in the inoculated plants (Fig. 2). The violet color is related to the synthesis of phenolic compounds that can limit oxidative stress levels and bind metals (Michalak 2006).

**Fig. 4:**
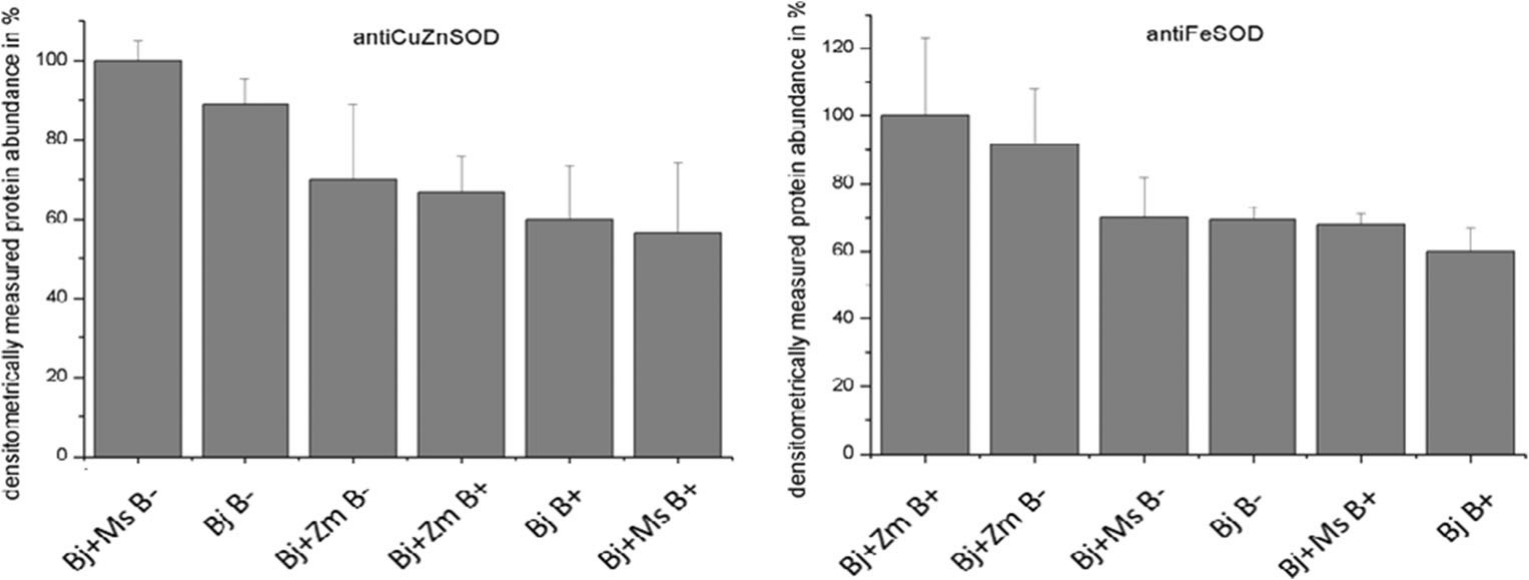
Superoxide dismutase isoforms level (CuZnSOD and FeSOD) in *B*. *juncea* plants grown in monoculture (Bj), with *Zea mays* (Bj + Zm) or with *Medicago sativa* (Bj + Ms), without (B−) or after *Burkholderia phytofirmans* inoculation (B+), detected using Western blot. Bj - *B*. *juncea*, Ms *- M*. *sativa*, Zm - *Z*. *mays*, “B−” - without bacterial inoculation, “B+” - inoculated plants. Mean values of three replicates (± SD)

In *B*. *juncea* plants inoculated with PGPR, compared with non-inoculated plants from the corresponding variants (independent cultivation, co-planting with *M*. *sativa*), a decrease in the level of synthesis of antioxidant enzymes (CuZnSOD) was also observed (Fig. 4). Interestingly, in the study of Peinado-Guevara et al. (2017) on *Solanum lycopersicum* grown with arbuscular mycorrhizal fungus (AMF) *Rhizophagus irregularis*, the authors also noted a decrease in CuZnSOD content after inoculation, with a simultaneous increase in ROS generation. The authors even hypothesized that genotypes displaying an increase in ROS concentration in leaves as a consequence of the decrease in antioxidative enzymes can trigger mycorrhiza-induced defenses. Our results could suggest that a similar mechanism is present after PGPR inoculation.

### Influence of PGPR and co-planting on efficiency of trace metals phytoextraction

Based on the values of the five observed parameters: Zn, Cd, Pb content, the number of collected plants (indicating survival), and the average dry biomass of plants, results of metal phytoextraction were collated made (Tab. 2). One of the main factors influencing the efficiency of phytoextraction is the high yield of dry biomass. *B*. *juncea*, characterized by a higher biomass production, is considered to be more efficient in Zn phytoextraction even than *T*. *caerulescens*, although it accumulates three times less Zn per kilogram of biomass compared with the hyperaccumulator (Bhargava et al. 2012). Despite the reduced number of plants, the average dry biomass of *B*. *juncea* from the variant of the “Bj + Zm” culture was increased in relation to the plant parameters from the “Bj” control variant (Fig. 2). It is worth paying attention to a very interesting observation that despite the reduction in the number of *B*. *juncea* seeds in the co-planting variants (from 12 to 6 seeds) and limiting the number of plants in the pot (up to 6 or 3), relative to the cultivation of *B*. *juncea* alone, the amount of collected plants (Tab. 2) in co-planting was 50% higher than in independent variants. It was respectively 80 and 69% (*B*. *juncea* cultivated with *M*. *sativa* without and after inoculation) and 61 and 56% (*B*. *juncea* cultivated with *Z*. *mays*, without and after inoculation), for independent cultivation without and after inoculation. In addition, crops from co-planting variants were characterized by higher average dry biomass and (in some cases) higher metal accumulation. Wu et al. (2006) also noted that PGPB inoculation indirectly translates into a higher efficiency of phytoextraction (higher uptake of metals) by increasing the dry biomass. On the other hand, the positive effect of co-planting cultures on yield is most probably related to, among others, the increase in the bioavailability of micro- and macroelements (Hauggaard-Nielsen et al. 2001), including trace metals. Thus, it was possible to confirm the hypothesis that the yield of metals from co-planting culture may be similar to that in independent culture, due to the better growth of the plants compared with monoculture (Jiang et al. 2010).

In the co-planting culture of *B*. *juncea* and *Z*. *mays*, the most important factor increasing the hypothetical efficiency of phytoextraction was the increase in the dry biomass of *Z*. *mays*. It is known that on soil with low availability of iron, *Z*. *mays* secretes phytosiderophores (Curie et al. 2009).

However, in the soil used for research, the level of bioavailable iron was high (Table 1), so there was no effect of co-planting on increasing metal uptake.

The influence of plants grown in co-planting cultures is difficult to classify, because it largely depends on the physicochemical properties of the soil. Similarly, Jiang et al. (2010) showed that in the conditions of hydroponic cultivation of *Z*. *mays*, independently and co-planted with the hyperaccumulator *Sedum alfredii* species, the factor modulating the uptake of metals was *S*. *alfredii* exudates, not *Z*. *mays* exudates. In the co-planting culture of *B*. *juncea* with *M*. *sativa*, the most significant impact on the hypothetical efficiency of phytoextraction was both the increase of metals in plants and the increase in the dry biomass. In a mesocosm experiment in which tobacco and clover were grown alongside, Liu et al. (2011) showed the relationship between co-planting and pH reduction that increased Cd mobility and BCFCd. Because the clover is closely related to *M*. *sativa*, it is possible that the results of the experiment presented in the current paper—an elevated level of metals in *B*. *juncea* plants cultivated with *M*. *sativa* (Fig.1)—could be explained partially by lowering the pH of *M*. *sativa*, resulting in increased availability of metals.

The presence of PGPR contributed to an increase in the dry biomass of *Z*. *mays* and *M*. *sativa* plants, relative to non-inoculated plants for each variant of the culture with *B*. *juncea* (Fig. 2). An increasing level of dry biomass is a frequent effect of PGPR inoculation. As shown by Upadhyay et al. (2012), wheat inoculation increases the level of dry biomass, total soluble sugar, and proline content. Similarly, Wu et al. (2006) observed that inoculation of *B*. *juncea* with PGPR (*Azotobacter chroococcum*, *Bacillus megaterium*, *Bacillus mucilaginosus*) protects plants from the effects of heavy metals and results in an increase in the dry biomass of plants. Sandhya et al. (2010) also observed that in PGPR-inoculated plants there is an increase in biomass, relative water content, leaf water potential, and mean stem diameter and a higher level of proline, sugars, and free amino acids. In the case of *B*. *juncea* plants, the increase in the harvest of dry biomass was influenced both by the cultivation of co-planting and the PGPR inoculation. Here, as in the case of the analyzed level of ROS and enzyme activity, the only exception to this profile were the *B*. *juncea* plants co-planted with the *Z*. *mays* after inoculation (relative to non-inoculated plants). In this variant, a reduced level of average dry biomass and a different chlorophyll *a* and *b* profile were observed (Fig. 2). This indicates additional interactions between these three organisms, but the explanation of this mechanism requires further research. Interestingly, *Z*. *mays* plants from this variant were characterized by increased average dry biomass, shoot length, and fresh biomass (Fig. 2). Jiang et al. (2008) studied the effect of *Burkholderia* on individual cultures of maize, Indian mustard, and tomato on soil contaminated with heavy metals: Pb (150.1 mg kg^−1^ of soil) and Cd (37.3 mg kg^−1^ of soil). The results indicated that inoculation resulted in an increase in the dry mass of *Z*. *mays* roots and shoots (by 75% and 30%, respectively) and Cd and Pb uptake, whereas in *B*. *juncea*, no significant increase was observed (except for the increased of Cd uptake in *B*. *juncea* roots). This may indicate the existence of a potentially lower positive effect of inoculation with *Burkholderia* on *B*. *juncea* compared with *Z*. *mays* plants. In our study, it was found that neither co-planting culture nor bacterial inoculation separately had any effect on the photosynthetic apparatus of *B*. *juncea* leaves, whereas their combined effect led to a significant decrease in the content of photosynthetic pigments (chlorophylls *a* and *b*) only in variants Bj + Ms B+. The effect of heavy metals on photosynthesis is quite widely reported in the scientific literature (Tran and Popova 2013; Muratova et al. 2015; Sitko et al. 2017). It is known that cadmium destroys the structure and function of chloroplasts, as well as reduces the content and ratio of photosynthetic pigments as a consequence of inhibition of the biosynthesis and degradation of chlorophyll (Muratova et al. 2015). It is not known what effect probiotic bacteria have on the photosynthetic apparatus. There are works that report that inoculation of stressed plants with plant growth–promoting microorganisms, e.g., *Rhizobium* sp., *Bacillus subtilis*, and *Pseudomonas fluorescens*, resulted in an increase in chlorophyll and carotenoid content (Wani and Khan 2013; Muratova et al. 2015). In the variant containing *B*. *juncea* with *M*. *sativa* and PGPR, it is possible that the bacterial inoculum increased heavy metal uptake, which was followed by an increase in the toxic effect of the metal on the photosynthetic apparatus. This explanation may be supported by the data demonstrating an enhancement of heavy metal accumulation by variant Bj + Ms B+.

## Conclusions

Our results show that the combined effect of co-planting and PGPR inoculation can increase the efficiency of phytoextraction. Optimization of the culture parameters: inoculation density, selection of accompanying plant species, PGPR strains, has the power to increase dry biomass yield and survivability and modulate the stress response and stress propagation in plants. The obtained results indicate that co-planting and PGPR inoculation have a positive effect on the phytoextraction process. We have shown an increase in the quantity and biomass of *B*. *juncea* in co-planting by over 50% compared with monoculture. Therefore, the use of co-planting in induced phytoextraction is of great significance for application.

Thus, the phytoextraction efficiency of these plants in large-scale crops and in the presence of PGPR bacteria should be checked. What is important from our point of view is the fact of monitoring soil microorganisms and their activity in assessing the effectiveness of the applied remediation method.

### Outlooks

The phytoextraction efficiency of these plants in large-scale crops and in the presence of PGPR bacteria should be checked. At the same time, the development of various bacterial consortia that would increase the accumulation of heavy metals in different soil conditions and for different plants would be of great practical importance.

## Electronic supplementary material

ESM 1 (DOC 154 kb).

## References

[CR1] Aebi HE, HU BERGMEYER (1983). Catalase. Methods of enzymatic analysis.

[CR2] Aelion CM, Davis HT (2007). Use of a general toxicity test to predict heavy metal concentrations in residential soils. Chemosphere.

[CR3] Ali H, Khan E, Sajad MA (2013). Phytoremediation of heavy metals—concepts and applications. Chemosphere.

[CR4] Andersen E, Peiter E, Küpper H (2018). Trace metal metabolism in plants. J Exp Bot.

[CR5] Arshad M, Saleem M, Hussain S (2007). Perspectives of bacterial ACC deaminase in phytoremediation. Trends Biotechnol.

[CR6] Babula P, Adam V, Havel L, Kizek R (2012). Cadmium accumulation by plants of *Brassicaceae* family and its connection with their primary and secondary metabolism. The plant family *Brassicaceae*.

[CR7] Beauchamp C, Fridovich I (1971). Superoxide dismutase: improved assays and an assay applicable to acrylamide gels. Anal Biochem.

[CR8] Belimov AA, Hontzeas N, Safronova VI, Demchinskaya SV, Piluzza G, Bullitta S, Glick BR (2005). Cadmium-tolerant plant growth-promoting bacteria associated with the roots of Indian mustard (Brassica juncea L. Czern.). Soil Biol Biochem.

[CR9] Bhargava A, Carmona FF, Bhargava M, Srivastava S (2012). Approaches for enhanced phytoextraction of heavy metals. J Environ Manag.

[CR10] Bhattacharyya PN, Jha DK (2012). Plant growth-promoting rhizobacteria (PGPR): emergence in agriculture. World J Microbiol Biotech.

[CR11] Bradford MM (1976). A rapid and sensitive method for the quantitation of microgram quantities of protein utilizing the principle of protein-dye binding. Anal Biochem.

[CR12] Chiang PN, Wang MK, Chiu CY, Chou SY (2006). Effects of cadmium amendments on low-molecular-weight organic acid exudates in rhizosphere soils of tobacco and sunflower. Environ Toxicol.

[CR13] Compant S, Kaplan H, Sessitsch A, Nowak J, Barka E A, Clément C (2008) Endophytic colonization of Vitis vinifera L. by Burkholderia phytofirmans strain PsJN: from the rhizosphere to inflorescence tissues. FEMS Microbiol Ecol 63(1):84–93. 10.1111/j.1574-6941.2007.00410.x 10.1111/j.1574-6941.2007.00410.x 18081592

[CR14] Curie C, Cassin G, Couch D, Divol F, Higuchi K, Le Jean M, Misson J, Schikora A, Czernic P, Mari S (2009). Metal movement within the plant: contribution of nicotianamine and yellow stripe 1-like transporters. Ann Bot.

[CR15] Doke N (1983). Involvement of superoxide anion generation in the hypersensitive response of potato tuber tissues to infection with an incompatible race of Phytophthora infestans and to the hyphal wall components. Physiol Plant Pathol.

[CR16] Douay F, Pelfrêne A, Planque J, Fourrier H, Richard A, Roussel H, Girondelot B (2013). Assessment of potential health risk for inhabitants living near a former lead smelter. Part 1: metal concentrations in soils, agricultural crops, and homegrown vegetables. Environ Monit Assess.

[CR17] Duarte B, Delgado M, Cacador I (2007). The role of citric acid in cadmium and nickel uptake and translocation, in Halimione portulacoides. Chemosphere.

[CR18] Etesami H (2018). Bacterial mediated alleviation of heavy metal stress and decreased accumulation of metals in plant tissues: mechanisms and future prospects. Ecotoxicol Environ Saf.

[CR19] Evangelou MWH, Ebel M, Schaeffer A (2006). Evaluation of the effect of small organic acids on phytoextraction of Cu and Pb from soil with tobacco *Nicotiana tabacum*. Chemosphere.

[CR20] Glick BR (2003). Phytoremediation: synergistic use of plants and bacteria to clean up the environment. Biotechnol Adv.

[CR21] Glick BR (2010). Using soil bacteria to facilitate phytoremediation. Biotechnol Adv.

[CR22] Goswami D, Thakker JN, Dhandhukia PC (2016). Portraying mechanics of plant growth promoting rhizobacteria (PGPR): a review. Cogent Food Agric.

[CR23] Guo HJ, Luo SL, Chen L, Xiao X, Xi Q, Wei WZ, Zeng G, Liu C, Wan Y, Chen J, He Y (2010). Bioremediation of heavy metals by growing hyperaccumulator endophytic bacterium Bacillus sp. L14. Bioresour Technol.

[CR24] Hanć A, Małecka A, Kutrowska A, Bagniewska–Zadworna A, Tomaszewska B, Barałkiewicz D (2016). Direct analysis of elemental biodistribution in pea seedlings by LA-ICP-MS, EDX and confocal microscopy: imaging and quantification. Microchem J.

[CR25] Hauggaard-Nielsen H, Ambus P, Jensen ES (2001). Interspecific competition, N use and interference with weeds in pea–barley intercropping. Field Crops Res.

[CR26] He LY, Chen ZJ, Ren GD, Zhang YF, Qian M, Sheng XF (2009). Increased cadmium and lead uptake of a cadmium hyperaccumulator tomato by cadmium-resistant bacteria. Ecotoxicol Environ Saf.

[CR27] He HD, Ye ZH, Yang DJ, Yan JL, Xiao L, Zhong T (2013). Characterization of endophytic Rahnella sp. JN6 from Polygonum pubescens and its potential in promoting growth and Cd, Pb, Zn uptake by Brassica napus. Chemosphere.

[CR28] Held DW, Gonsiska P, Potter DA (2003). Evaluating companion planting and non-host masking odors for protecting roses from the Japanese beetle (Coleoptera: Scarabaeidae). J Econ Entomol.

[CR29] Jadia C, Fulekar M (2009) Phytoremediation of heavy metals: recent techniques. African J Biotech 8:921–928

[CR30] Jiang CY, Sheng XF, Qian M, Wang QY (2008) Isolation and characterization of a heavy metal-resistant *Burkholderia sp*. from heavy metal-contaminated paddy field soil and its potential in promoting plant growth and heavy metal accumulation in metal-polluted soil. Chemosphere 72(2):157–164. 10.1016/j.chemosphere.2008.02.006 10.1016/j.chemosphere.2008.02.006 18348897

[CR31] Jiang W, Liu D, Hou W (2000). Hyperaccumulation of lead by roots, hypocotyls, and shoots of *Brassica juncea*. Biol Plant.

[CR32] Jiang C, Wu QT, Sterckeman T, Schwartz C, Ouvrard S, Perriguey J, Morel JL (2010). Co-planting can phytoextract similar amounts of cadmium and zinc to mono-cropping from contaminated soils. Ecol Eng.

[CR33] Karami A, Shamsuddin Z (2010). Phytoremediation of heavy metals with several efficiency enhancer methods. Afr J Biotech.

[CR34] Khan AG (2005). Role of soil microbes in the rhizosphere of plants growing on trace metal contaminated soils in phytoremediation. J Trace Elem Med Biol.

[CR35] Kohler J, Hernández JA, Caravaca F, Roldán A (2009). Induction of antioxidant enzymes is involved in the greater effectiveness of a PGPR versus AM fungi with respect to increasing the tolerance of lettuce to severe salt stress. Environ Exp Bot.

[CR36] Komarek M, Tlustos P, Szakova J, Chrastny V, Ettler V (2007). The use of maize and poplar in chelant-enhanced phytoextraction of lead from contaminated agricultural soils. Chemosphere.

[CR37] Kramer U (2010). Metal hyperaccumulation in plants. Annu Rev Plant Biol.

[CR38] Kucharski R, Sas-Nowosielska A, Małkowski E, Japenga J, Kuperberg JM, Pogrzeba M, Krzyżak J (2005). The use of indigenous plant species and calcium phosphate for the stabilization of highly metal-polluted sites in southern Poland. Plant Soil.

[CR39] Kutrowska A, Małecka A, Piechalak A, Masiakowski W, Hanć A, Barałkiewicz D, Tomaszewska B (2017). Effects of binary metal combinations on zinc, copper, cadmium and lead uptake and distribution in *Brassica juncea*. J Trace Elem Med Biol.

[CR40] Leštan D, Luo CL, Li XD (2008). The use of chelating agents in the remediation of metal-contaminated soils: a review. Environ Poll.

[CR41] Li WC, Ye ZH, Wong MH (2007). Effects of bacteria on enhanced metal uptake of the Cd/Zn hyperaccumulating plant, Sedum alfredii. J Exp Bot.

[CR42] Liu L, Li Y, Tang J, Hu L, Chen X (2011). Plant coexistence can enhance phytoextraction of cadmium by tobacco (*Nicotiana tabacum* L.) in contaminated soil. J Environ Sci.

[CR43] Lopez ML, Peralta-Videa JR, Benitez T, Gardea-Torresdey JL (2005). Enhancement of lead uptake by alfalfa (*Medicago sativa*) using EDTA and a plant growth promoter. Chemosphere.

[CR44] Ma Y, Rajkumar M, Freitas H (2009). Inoculation of plant growth promoting bacterium *Achromobacter xylosoxidans* strain Ax10 for the improvement of copper phytoextraction by *Brassica juncea*. J Environ Manag.

[CR45] Ma Y, Rajkumar M, Rocha I, Oliveira RS, Freitas H (2015). Serpentine bacteria influence metal translocation and bioconcentration of Brassica juncea and Ricinus communis grown in multi-metal polluted soils. Front Plant Sci.

[CR46] Ma Y, Rajkumar M, Zhang C, Freitas H (2016). Beneficial role of bacterial endophytes in heavy metal phytoremediation. J Environ Manag.

[CR47] Markmann K, Parniske M (2009). Evolution of root endosymbiosis with bacteria: how novel are nodules?. Trends Plant Sci.

[CR48] Marques A, Rangel A, Castro PML (2009). Remediation of heavy metal contaminated soils: phytoremediation as a potentially promising clean-up technology. Crit Rev Environ Sci Technol.

[CR49] McGrath SP, Zhao FJ (2003). Phytoextraction of metals and metalloids from contaminated soils. Curr Opin Biotechnol.

[CR50] Mead R, Willey RW (1980). The concept of a ‘land equivalent ratio’ and advantages in yields from intercropping. Exp Agric.

[CR51] Meyers DE, Auchterlonie GJ, Webb RI, Wood B (2008). Uptake and localization of lead in the root system of *Brassica juncea*. Environ Pollut.

[CR52] Michalak A (2006). Phenolic compounds and their antioxidant activity in plants growing under heavy metal stress. Pol J Environ Stud.

[CR53] Muratova A, Lyubun Y, German K, Turkovskaya O (2015). Effect of cadmium stress and inoculation with a heavy-metal-resistant bacterium on the growth and enzyme activity of *Sorghum bicolour*. Environ Sci Pollut Res.

[CR54] Nakano Y, Asada K (1981). Hydrogen peroxide is scavenged by ascorbate-specific peroxidase in spinach chloroplasts. Plant Cell Physiol.

[CR55] Neill SJ, Desikan R, Clarke A, Hurst RD, Hancock JT (2002). Hydrogen peroxide and nitric oxide as signalling molecules in plants. J Exp Bot.

[CR56] Neugschwandtner RW, Tlustos P, Komarek M, Szakova J (2008). Phytoextraction of Pb and Cd from a contaminated agricultural soil using different EDTA application regimes: laboratory versus field scale measures of efficiency. Geoderma.

[CR57] Neuman DR, Schafer WM (2006) Phytostabilization of fluvial tailings deposits in the Clark Fork river floodplain. In Proc. Int. Conf. Acid Rock Drainage, 7th, St. Louis, pp 26-30

[CR58] Niu L, Shen Z, Luo C, Deng Y, Wang C (2012). Accumulation mechanisms and subcellular distribution of Cu in maize grown on soil treated with [S, S]-ethylenediamine disuccinic acid. Plant Soil.

[CR59] Olowe VIO, Adeyemo AY (2009). Enhanced crop productivity and compatibility through intercropping of sesame and sunflower varieties. Ann Appl Biol.

[CR60] Patterson BD, MacRae EA, Ferguson IB (1984). Estimation of hydrogen peroxide in plant extracts using titanium (IV). Anal Biochem.

[CR61] Peinado-Guevara LI, López-Meyer M, López-Valenzuela JA, Maldonado-Mendoza IE, Galindo-Flores H, Campista-León S, Medina-Godoy S (2017). Comparative proteomic analysis of leaf tissue from tomato plants colonized with *Rhizophagus irregularis*. Symbiosis.

[CR62] Penrose DM, Glick BR (2003). Methods for isolating and characterizing ACC deaminase-containing plant growth promoting rhizobacteria. Physiol Plant.

[CR63] Pillay VK, Nowak J (1997). Inoculum density, temperature, and genotype effects on in vitro growth promotion and epiphytic and endophytic colonization of tomato (*Lycopersicon esculentum* L.) seedlings inoculated with a pseudomonad bacterium. Can J Microbiol.

[CR64] Poupin MJ, Timmermann T, Vega A, Zuñiga A, González B (2013). Effects of the plant growth promoting bacterium *Burkholderia phytofirmans* PsJN throughout the life cycle of *Arabidopsis thaliana*. PLoS One.

[CR65] Prasad VMN, de Oliveira Freitas HM (2003). Metal hyperaccumulation in plants: biodiversity prospecting for phytoremediation technology. Electron J Biotech.

[CR66] Rajkumar M, Ae N, Prasad MNV, Freitas H (2010). Potential of siderophore-producing bacteria for improving heavy metal phytoextraction. Trends Biotechnol.

[CR67] Rajkumar M, Prasad MNV, Sandhya S, Freitas H (2013). Climate change driven plant-metal-microbe interactions. Environ Int.

[CR68] Rascio N, Navari-Izzo F (2011). Heavy metal hyperaccumulating plants: how and why do they do it? And what makes them so interesting?. Plant Sci.

[CR69] Robinson BH, Anderson CWN, Dickinson NM (2015). Phytoextraction: where’s the action?. J Geochem Explor.

[CR70] Ronen R, Galun M (1984). Pigment extraction from lichens with dimethyl sulfoxide (DMSO) and estimation of chlorophyll degradation. Environ Exp Bot.

[CR71] Saleem M, Arshad M, Hussain S, Bhatti AS (2007). Perspective of plant growth promoting rhizobacteria (PGPR) containing ACC deaminase in stress agriculture. J Ind Microbiol Biotechnol.

[CR72] Sandhya V, Ali SZ, Grover M, Reddy G, Venkateswarlu B (2010). Effect of plant growth promoting *Pseudomonas* spp. on compatible solutes, antioxidant status and plant growth of maize under drought stress. Plant Growth Regul.

[CR73] Sarma H (2011). Metal hyperaccumulation in plants: a review focusing on phytoremediation technology. J Environ Sci Technol.

[CR74] Sessitsch A, Coenye T, Sturz A V, Vandamme P, Barka E A, Salles J F, Van Elsas J D, Faure D, Reiter B, Glick B R, Pruski G, Nowak J (2005) *Burkholderia phytofirmans* sp. nov., a novel plant-associated bacterium with plant-beneficial properties. Inter J System Evol Microbiol 55:1187–1192. 10.1099/ijs.0.63149-0 10.1099/ijs.0.63149-0 15879253

[CR75] Sessitsch A, Kuffner M, Kidd P, Vangronsveld J, Wenzel WW, Fallmann K, Puschenreiter M (2013). The role of plant-associated bacteria in the mobilization and phytoextraction of trace elements in contaminated soils. Soil Biol Biochem.

[CR76] Sitko K, Rusionowski S, Kalaji HM, Szopiński M, Małkowski E (2017). Photosynthetic efficiency as bioindicator of environmental pressure in A. halleri. Plant Physiol.

[CR77] Slesak I, Libik M, Karpinska B, Karpinski S, Miszalski Z (2007). The role of hydrogen peroxide in regulation of plant metabolism and cellular signalling in response to environmental stresses. Acta Biochim Pol.

[CR78] Sobariu DL, Fertu DIT, Diaconu M, Pavel LV, Hlihor RM, Drăgoi EN, Curteanu S, Lenz M, Corvini PF, Gavrilescu M (2017). Rhizobacteria and plant symbiosis in heavy metal uptake and its implications for soil bioremediation. New Biotechnol.

[CR79] Srivastava S, Singh N (2014). Mitigation approach of arsenic toxicity in chickpea grown in arsenic amended soil with arsenic tolerant plant growth promoting Acinetobacter sp. Ecol Eng.

[CR80] Stuczyński T, Pistelok F, Siebielec G, Kukla H, Daniels W, Chaney R, Pantuck K (2000) Biological aspects of metal waste reclamation with sewage sludge in Poland. In Proceedings of the symposium on mining, forest and land restoration: the successful use of residuals/biosolids/organic matter for reclamation activities, Denver, July 17-20

[CR81] Temperton VM, Mwangi PN, Scherer-Lorenzen M, Schmid B, & Buchmann N (2007) Positive interactions between nitrogen-fixing legumes and four different neighbouring species in a biodiversity experiment. Oecologia 151(2):190–205 10.1007/s00442-006-0576-z 17048010

[CR82] Tiemann KJ, Gamez G, Dokken K, Parsons JG, Gardea-Torresdey JL (2002). Chemical modification and X-ray absorption studies for lead(II) binding by *Medicago sativa* (alfalfa) biomass. Microchem.

[CR83] Tran TA, Popova LP (2013). Functions and toxicity of cadmium in plants: recent advances and future prospects. Turk J Bot.

[CR84] Upadhyay SK, Singh JS, Saxena AK, Singh DP (2012). Impact of PGPR inoculation on growth and antioxidant status of wheat under saline conditions. Plant Biol.

[CR85] Vamerali T, Bandiera M, Mosca G (2010). Field crops for phytoremediation of metal-contaminated land. A review. Environ Chem Lett.

[CR86] Van Loon LC (2007). Plant responses to plant growth-promoting rhizobacteria. Eur J Plant Pathol.

[CR87] Verbruggen N, Hermans C, Schat H (2009). Molecular mechanisms of metal hyperaccumulation in plants. New Phytol.

[CR88] Verbruggen N, Juraniec M, Baliardini C, Meyer CL (2013). Tolerance to cadmium in plants: the special case of hyperaccumulators. Biometals.

[CR89] Wang Q, Li Y, Alva A (2010). Cropping systems to improve carbon sequestration for mitigation of climate change. J Environ Prot.

[CR90] Wani PA, Khan MS (2013). Nickel detoxification and plant growth promotion by multi metal resistant plant growth promoting *Rhizobium* species RL9. Bull Environ Contam Toxicol.

[CR91] Weilharter A, Mitter B, Shin MV, Chain PSG, Nowak J, Sessitsch A (2011). Complete genome sequence of the plant growth promoting endophyte *Burkholderia phytofirman*s strain PsJNT. J Bacteriol.

[CR92] Weyens N, van der Lelie D, Taghavi S, Vangronsveld J (2009). Phytoremediation: plant–endophyte partnerships take the challenge. Curr Opin Biotechnol.

[CR93] Wood JL, Tang C, Franks AE (2016). Microbial associated plant growth and heavy metal accumulation to improve phytoextraction of contaminated soils. Soil Biol Biochem.

[CR94] Wu CH, Wood TK, Mulchandani A, Chen W (2006). Engineering plant-microbe symbiosis for rhizoremediation of heavy metals. Appl Environ Microbiol.

[CR95] Wu QT, Wei ZB, Ouyang Y (2007). Phytoextraction of metal-contaminated soil by *Sedum alfredii* H: effects of chelator and coplanting. Water Air Soil Poll.

[CR96] Yang J, Kloepper JW, Ryu C-M (2009). Rhizosphere bacteria help plants tolerate abiotic stress. Trends Plant Sci.

[CR97] Yang R, Liu L, Zan S, Tang J, Chen X (2012). Plants species coexistence alleviates the impacts of lead on *Zea mays* L. J Environ Sci.

[CR98] Zhao Z, Xi M, Jiang G, Liu X, Bai Z, Huang Y (2010). Effects of IDSA, EDDS and EDTA on heavy metals accumulation in hydroponically grown maize (*Zea mays*, L.). J Hazard Mater.

