# Supplementary material for: Combined use of companion planting and PGPR for the assisted phytoextraction of trace metals (Zn, Pb, Cd)
Source: Environ Sci Pollut Res Int. 2020 Feb 8;27(12):13809–25. doi: 10.1007/s11356-020-07885-3 (PMC7162837; doi:10.1007/s11356-020-07885-3)
Supplement: Supplementary file 1 — (DOC 154 kb). [file 11356_2020_7885_MOESM1_ESM.doc]

*Environmental Science and Pollution Research*

**Combined use of companion planting and PGPR for the assisted phytoextraction of trace metals (Zn, Pb, Cd)**

Agnieszka Konkolewska^1^, Aneta Piechalak^2^, Liliana Ciszewska^1^, Nina Antos-Krzemińska^3^, Tomasz Skrzypczak^4^, Anetta Hanć^5^, Krzysztof Sitko^6^, Eugeniusz Małkowski^6^, Danuta Barałkiewicz^5^, Arleta Małecka^7^

^1^Department of Biochemistry, ^2^Department of Genom Biology, ^3^Department of Bioenergetics, ^4^Department of Molecular and Cellular Biology, ^7^Department of Biotechnology, Institute of Molecular Biology and Biotechnology, Adam Mickiewicz University in Poznan, Uniwersytet Poznanski 6 street, 61-614 Poznan, Poland

of ^5^Department of Trace Element Analysis by Spectroscopy Method, Faculty Chemistry, Adam Mickiewicz University in Poznań, Uniwersytet Poznanski 8 street, 61-614 Poznan, Poland

^6^Department of Plant Physiology, Faculty of Biology and Environmental Protection, University of Silesia in Katowice, 40-032 Katowice, Poland

Corresponding author: Agnieszka Kutrowska (e-mail: [akutr@amu.edu.pl](mailto:akutr@amu.edu.pl)); Arleta Małecka (e-mail:arletam@amu.edu.pl).

#### Table S1 ICP operating conditions

| **ICP-MS** |  |
| --- | --- |
| Instrument | PE Sciex ELAN 6100 DRC II |
| Nebulizer gas flow, L/min | 0.86-0.9 |
| Auxiliary gas flow, L/min | 0.8 |
| Plasma gas flow, L/min | 15 |
| RF power, W | 1100 |
| Lens setting | autolens calibrated |
| Detector mode | dual (pulse counting and analogue mode) |
| Sweeps/Reading/Replicate | 2 / 180 / 1 |
| Measured mass | ^111^Cd, ^65^Cu, ^208^Pb, ^66^Zn |
| Internal standard |  |
| **LASER ABLATION** |  |
| Laser ablation system | CETAC LSX-500, Nd:YAG |
| Wavelength, nm | 266 |
| RF power, W 1100 | 1250 |
| Nebulizer gas flow, L/min | 0.95-1.05 |
| Pulse duration, ns | 5 |
| Ablation frequency, Hz | 10 |
| Spot size, µm | 50 |
| Laser energy, mJ | 8.1 |
| Scan rate, µm/s | 25 |
| Scan method | single line scan; peak hoping |
| Internal standard | ^13^C |

Whilst tuning the ICP-MS, compromise conditions for maximum signal intensity of the analyte (^24^Mg^+^, ^115^In^+^, ^238^U^+^) and minimum ratio of oxide (^140^Ce^16^O^+^/^140^Ce<3%) and doubly charged ions (^128^Ba^2+^/^128^Ba^+^<3%) were found. The proper working conditions of ICP-MS working were checked by using a solution containing Mg, In, U at concentration of 1 mg L-1 and Ba concentration of 10 mg L-1 (Smart Tune Solution e Elan DRC II/plus, Atomic Spectroscopy Standard, Perkin Elmer Pure). Calibrations curves were established using aqueous standards of Cd, Cu, Pb and Zn. A mixed standard solution (Multielement Calibration Standard 3, Atomic Spectroscopy Standard, PerkinElmer Pure) containing analyzed elements at a concentration of 10 mg L^-1^ was used to construct calibration curves. The calibration curves for the elements were constructed in the range of 0.01 to 50 μgL^-1^. The isotopes of ^45^Sc, ^74^Ge, ^103^Rh, and ^159^Tb prepared from individual solutions with a concentration of 1000 mg L^-1^ were applied as internal standards (ICP Standard CertiPUR, Merck, Germany). The internal standards concentration of 10 µg L^-1^, allowed to eliminate non spectral interferences and instrumental drift. In turn, spectral interferences were eliminated through the use of a dynamic reaction cell (DRC).

Laser performance was optimized according to a detailed scheme (Hanć, Olszewska, and Baralkiewicz 2013) using a single variable method. A final selection of parameters was based on maximum sensitivity and most stable signal intensities. During the analysis of the samples intensity of the analytical signal of ^13^C as an internal standard was registered. Carbon isotope was chosen as an internal standard, because it is connected with the sample's matrix and compensates the differences in the amount of sample's material being ablated. Application of ^13^C as internal standard for soft tissues analysis was possibly because signal from the samples was greater than 6% of the total ^13^C signal (Austin *et al.* 2011, Hanć *et al.* 2014).

**Analytical Performance**

#### After calibration, and also during the analysis, measurements were controlled by analysis of standard solutions at concentrations of 1μgL^-1^ or 5μgL^-1^ and certified reference materials after each batch of fifteen samples. The calibration curves for determined elements were linear in the range of calibration standards. The correlation coefficient R exceeded a value of 0.999 The trueness of the analytical results was assessed using the reference material NIST SRM 1515 Apple Leaves and NIST SRM Spinach Leaves 1575a. The accuracy of the method for investigated elements was evaluated by determining the percentage bias between the measured concentration of the applied certified reference materials (CRMs) and its certified value. The bias represents the difference between the CRM elemental concentration measured using ICP-MS and the certified value, which is: 1.5% for Cd, 2.3% for Cu, 1.7% for Pb, and 2.5% for Zn. The limits of detection (LOD) for determined elements were counted according to LOD=3.3 S/b, where S means standard deviation of the result obtained for the blank samples and b is the sensitivity. The LODs for the ICP-MS method were found to be 0.02 µg g^-1^(Cd), 0.05 µg g^-1^ (Cu), 0.008 µg g^-1^ (Pb) and 0.01 mg g^-1^ (Zn). LOQ values were calculated as three times of the LOD values. Precision was calculated as the relative standard deviation expressed as %. As a result of the analysis, the precision values were calculated for Cd (1,2%), Cu (2,8%), Pb (1,7%) and Zn (2,4%).


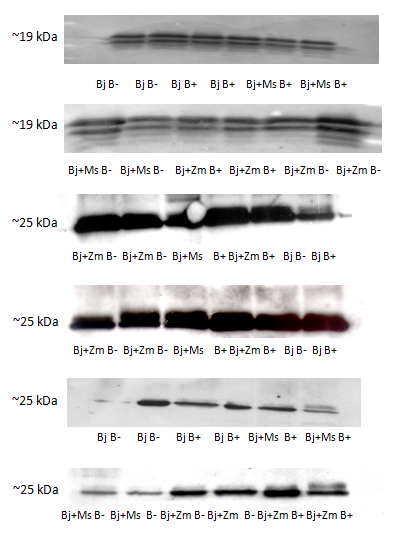


Fig.S1 Representative blots for antiCuZnSOD (part A) and antiFeSOD antibody (part B), (50 µg of protein per well).
